# Transmembrane adaptor protein PAG is a mediator of PD-1 inhibitory signaling in human T cells

**DOI:** 10.1038/s42003-021-02225-8

**Published:** 2021-06-03

**Authors:** Marianne Strazza, Inbar Azoulay-Alfaguter, Michael Peled, Kieran Adam, Adam Mor

**Affiliations:** 1 Columbia Center for Translational Immunology, Columbia University Medical Center, New York, NY USA; 2 Perlmutter Cancer Center, New York University School of Medicine, New York, NY USA; 3 Division of Rheumatology, Department of Medicine, Columbia University Medical Center, New York, NY USA

**Keywords:** Tumour immunology, Cell signalling

## Abstract

The inhibitory receptor PD-1 is expressed on T cells to inhibit select functions when ligated. The complete signaling mechanism downstream of PD-1 has yet to be uncovered. Here, we discovered phosphoprotein associated with glycosphingolipid-enriched microdomains 1 (PAG) is phosphorylated following PD-1 ligation and associate this with inhibitory T cell function. Clinical cohort analysis correlates low PAG expression with increased survival from numerous tumor types. PAG knockdown in T cells prevents PD-1-mediated inhibition of cytokine secretion, cell adhesion, CD69 expression, and ERK_204/187_ phosphorylation, and enhances phosphorylation of SRC_527_ following PD-1 ligation. PAG overexpression rescues these effects. In vivo, PAG contributes greatly to the growth of two murine tumors, MC38 and B16, and limits T cell presence within the tumor. Moreover, PAG deletion sensitizes tumors to PD-1 blockade. Here PAG is established as a critical mediator of PD-1 signaling and as a potential target to enhance T cell activation in tumors.

## Abstract (web summary)

Strazza et al. show that phosphoprotein associated with glycosphingolipidenriched microdomains 1 (PAG) is phosphorylated following PD-1 ligation. They find that PAG deletion sensitizes tumors to PD-1 blockade. This study suggests PAG as a critical mediator of PD-1 signaling and a promising target to enhance T cell activation in tumors.

## Introduction

An effective T cell response to tumors is essential for limiting tumor growth and increasing overall survival. Antigen-specific T cells must be activated and present within the tumor in order to mediate tumor regression. Surface expression of the inhibitory receptor PD-1 is upregulated on activated T cells^1^, allowing for enhanced signaling in response to its ligands PD-L1 or PD-L2 being expressed on other cells within the tumor microenvironment, and therefore increased inhibition of numerous T cells functions. For these reasons, immune checkpoint inhibitors, including PD-1-targeting antibodies, are proving successful in the treatment of diverse tumors.

Despite the success of anti-PD-1 therapeutics, there is room for improvement and innovation within the field of checkpoint inhibitors. The overall response rate to PD-1 blockade ranges from 11 to 65%, averaging at 23% for most type of tumors^2,3^. One approach to improving efficacy lies in identifying downstream signaling mediators that are essential for the inhibitory effects of PD-1 ligation. While the functional consequences of PD-1 signaling and blockade are well-studied, the molecular signaling mechanisms remain poorly characterized. The major known mediator of PD-1 signaling is the tyrosine phosphatase SHP-2^4,5^, though we have shown that numerous proteins are phosphorylated following PDL1/PDL2 binding^6^. Multiple recent studies have questioned the role of SHP-2 in this pathway by demonstrating that this phosphatase was dispensable for PD-1 signaling^7^. This suggests that there is more complexity to PD-1 signaling within T cells than is currently understood.

Immunoreceptor signaling is propagated through the recruitment of cytoplasmic signaling or effector proteins with SH2 domains to the plasma membrane, suggesting the need for membrane-associated proteins with phosphotyrosines that bind SH2 domains. A family of proteins that fills this role is the transmembrane adapter proteins (TRAP). Members of the TRAP family have short extracellular domains and long cytoplasmic domains containing a variable number of tyrosine residues^8^. Notably, none of the identified TRAP family members contain a classical immunoreceptor tyrosine-based activation motif (ITAM)^8^. Phosphoprotein associated with glycosphingolipid-enriched microdomains 1 (PAG) is a member of the TRAP family. The cytoplasmic tail of PAG contains ten tyrosine residues, eight of which have been reported to be phosphorylated^8^. One of these phosphotyrosines is a binding site for the protein tyrosine kinase C-terminal SRC kinase (CSK)^8,9^, a negative regulator of the SRC family, and another is a binding site for the inhibitory protein RasGAP^10^. At resting state, PAG is one of the more tyrosine-phosphorylated membrane proteins in T cells^8,9^. Following stimulation through the T cell receptor (TCR), PAG is dephosphorylated^8,9,11–13^^,^ allowing for the release of CSK from the membrane and the release of SRC inhibition. We have found that three tyrosines in the cytoplasmic tail of PAG are phosphorylated following PD-1 ligation, which led us to the hypothesis that PAG is an effector downstream of PD-1 that contributes to the inhibitory function of PD-1 in T cells. To test this hypothesis, we generated PAG-deficient and PAG-overexpressing T cells, and utilized PAG knockout mice to interrogate the contributions of PAG to PD-1-driven T cell inhibition.

## Results

### PAG is associated with negative regulation of biological processes

**Fig. 1:**
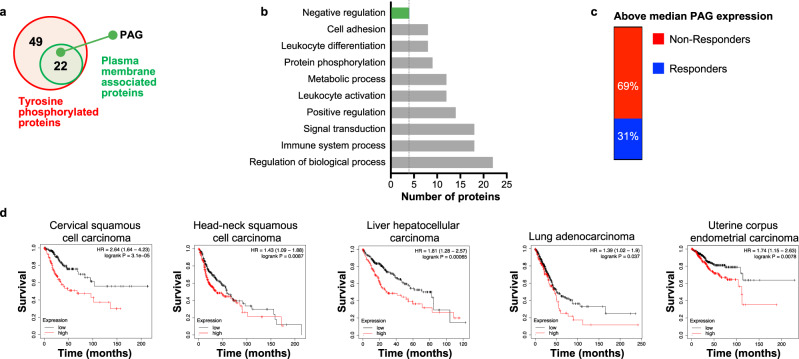
PAG is associated with negative regulation of biological processes. **a** Phosphoproteins were enriched for in the lysates of anti-CD3 or anti-CD3 + PDL2 stimulated Jurkat cells then identified by mass-spec^30^. 49 proteins were found to be enriched in the anti-CD3 + PDL2 condition relative to unstimulated or anti-CD3. 22 of these proteins were plasma membrane associated, including PAG. **b** Analysis of the biological processes associated with the 22 plasma membrane associated phosphoproteins. PAG is associated with negative regulation of biological processes (green bar). **c** Single cell analysis of PAG expression in T cells isolated from cancer patients that received therapeutic PD-1 blockade. Samples are categorized based on patient response to PD-1 blockade (responder eight patients—5110 cells/non-responder 18 patients—10,190 cells) and by median PAG expression level. PAG expression displayed is upper 50%. **d** TCGA data displayed to show overall survival of high and low PAG expression defined by median expression for each cohort. *p < 0.05.

To identify potential downstream mediators of PD-1 signaling in T cells, we enriched cell lysates for phosphorylated proteins following stimulation with anti-CD3 alone or in combination with PDL2 and analyzed them by mass spectrometry^6^. We found 49 proteins with phosphotyrosines that were more prevalent in T cells stimulated with anti-CD3 and PDL2 in combination, and 22 of these 49 proteins are plasma membrane associated (Fig. 1a and Table 1). The conditions of this analysis suggest that these tyrosine residues are phosphorylated following anti-CD3 and PDL2 stimulation.

**Table 1:** Plasma membrane proteins that were phosphorylated downstream of PD-1 signaling.

Uni Prot	Gene	pY	adj. *p* val.
Q99704	DOK1	362	0.001
P07355	ANXA2	24	0.001
P07766	CD3E	188, 199	0.001
P16284	PECAM1	713	0.001
P43403	ZAP70	292, 492	0.001
P29350	PTPN6	536, 564	0.001
Q9UJU6	DBNL	344	0.001
P06239	LCK	394, 505	0.001
P10747	CD28	191, 209	0.001
P20963	CD247	142	0.002
Q9HBG7	LY9	603	0.002
Q13546	RIPK1	384	0.004
P04234	CD3D	149	0.005
O43561	LAT	110	0.005
O75185	ATP2C2	131	0.008
P14317	HCLS1	140	0.008
Q9NWQ8	PAG1	181, 317, 417	0.009
Q96DU3	SLAMF6	309	0.021
Q8TF74	WIPF2	74	0.034
P62136	PPP1CA	306	0.034
Q9UIBB	CD84	316	0.043
Q13009	TIAM1	829	0.043

We found PAG included in the 22 plasma membrane associated proteins that have increased phosphorylation of select tyrosine residues. PAG is expressed in hematopoietic cells, with high expression in CD4^+^ and CD8^+^ T cells^6^ (Supplementary Fig. 1). PAG has ten tyrosines in its cytoplasmic tail. We have found three of these tyrosines, Y181 Y317, and Y417, to be phosphorylated following PD-1 signaling (Table 1)^6^. We additionally demonstrated that PAG is phosphorylated downstream of both PDL1 and PDL2 signaling (Supplementary Fig. 2). PAG is phosphophorylated in resting T cells by Fyn at Y317 and binds cytosolic Csk, anchoring the kinase near the membrane. Csk in turn phosphorylates C-termini inhibitory tyrosines of Src kinases maintaining an inhibitory state^14^. Early TCR signaling events lead to PAG Y317 dephosphorylation and release of Csk binding, and phosphodeficient mutation of this position (Y317F) abolishes Csk binding^9^. PAG also negatively regulates Ras by binding RasGAP at PAG Y181 and anchoring the GTPase activating protein at the plasma membrane^14^. Phosphodeficient mutation of Y181 disrupts RasGAP binding, though disrupting both tyrosines at 181 and 317 are necessary to interfere with inhibition of Ras^15^. The contribution of Y417 to PAG function is not well defined, though it has been shown that phosphodeficient mutation of this position (Y417F) has no effect on interaction with Fyn or Csk^9^.

Analysis of the GO Biological Processes^16,17^ distribution of the 22 plasma membrane associated proteins demonstrates that these proteins contribute to diverse cellular functions (Fig. 1b). The proteins PTPN6, PAG, RIPK1, and SLAMF5 were associated with negative biological processes. Of particular interest to us, PAG was associated with negative regulation of cellular function. Phosphorylation of PAG downstream of PD-1 ligation suggests that PAG may contribute to mediating PD-1 inhibition of T cell function.

It has been previously shown that single nucleotide polymorphisms (SNP) in the non-coding region of PAG that correlate with lower PAG protein expression also correlate with better overall survival in cutaneous melanoma patients^18^. A single cell analysis of T cells isolated from cancer patients that have undergone PD-1 blockade therapy shows that high expression of PAG is correlated with unresponsiveness to PD-1 blockade (Fig. 1c). Through analysis of the samples included in The Cancer Genome Atlas (TCGA)^2,19^, we found that low PAG expression is correlated with improved survival with multiple tumor types (Fig. 1d). This inverse correlation is consistent with an inhibitory role in T cells.

### PAG is required for PD-1 signaling and function

**Fig. 2:**
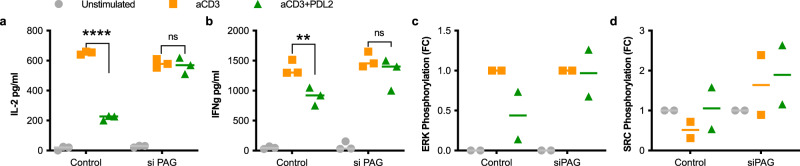
PAG contributes to PD-1 signaling in primary T cells. **a**, **b** ELISA of secreted IL-2 (**a**) and IFNγ (**b**) in the supernatants of primary human CD3^+^ T cells collected 48 h following stimulation by magnetic beads. T cells either expressed a non-targeting siRNA (control) or PAG targeting siRNA pool (siPAG). **c**, **d** Phosphorylated ERK (**c**) and phosphorylated SRC (**d**) were detected by western blot of human CD3^+^ T cell lysates 5 min after stimulation by magnetic beads. Fold change is calculated relative to anti-CD3 stimulation (**c**) or unstimulated (**d**). Individual data points shown with median indicated of two or three independent experiments. **p < 0.01; ****p < 0.0001; ns not significant.

To characterize the role of PAG in PD-1 inhibition of T cell function we depleted the levels of PAG expressed in primary human or Jurkat T cells (Supplementary Fig. 3A). We observed that IL-2 and IFNγ secretion was increased by stimulation with anti-CD3 antibody, as expected (Fig. 2a, b and Supplementary Figs. 4, and 5a, b). While this increase was inhibited by the introduction of PDL1 or PDL2 in wild type primary human T cells, the depletion of PAG prevented any inhibition of IL-2 or IFNγ secretion by PDL2 (Fig. 2a, b and Supplementary Figs. 4, and 5a, b). ERK phosphorylation is increased following anti-CD3 stimulation, and inhibition by PDL2 that is dependent on PAG expression in primary human T cells (Fig. 2c and Supplementary Figs. 4b and 5c). SRC kinase is regulated by phosphorylation and dephosphorylation, with an inhibiting phosphotyrosine at position 527. Dephosphorylation of tyrosine 527 following stimulation with anti-CD3 (Fig. 2d and Supplementary Figs. 4c and 5d) increases SRC kinase activity. Phosphorylation of tyrosine 527 is high with the introduction of PDL2 in primary human T cells, but is diminished in PAG depleted cells (Fig. 2d and Supplementary Figs. 4c and 5d).

**Fig. 3:**
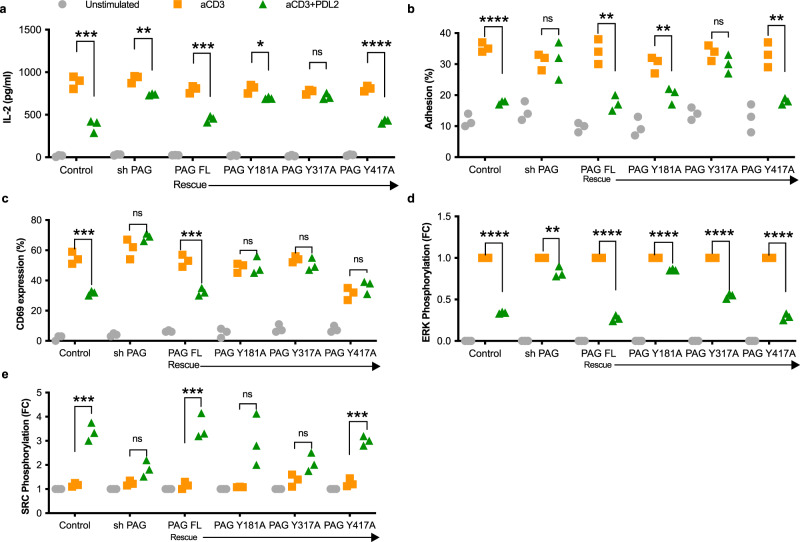
PAG is required for PD-1 signaling and function. **a** ELISA of secreted IL-2 in the supernatants of Jurkat cells collected 24 h following stimulation by magnetic beads. Jurkat T cells either expressed a non-targeting shRNA (control) or PAG targeting shRNA (shPAG). For rescue transfections, shPAG Jurkats were transiently transfected with full length, wild type PAG (PAG FL) or with phosphodeficient mutants (Y181A, Y317A, and Y417A). **b** Adhesion assay of Jurkat cells to fibronectin following stimulation for 15 min. The number of adherent cells remaining, expressed as a percentage of the total number of labeled cells, was determined with a fluorescent plate reader. PAG knockdown and rescue transfections as in **a**. **c** Percentage of Jurkat cells expressing CD69 on the surface following 24-hour stimulation, measured by flow cytometry. PAG knockdown and rescue transfections as in **a**. **d**, **e** Phosphorylated ERK (**d**) and phosphorylated SRC (**e**) were detected by western blot of Jurkat lysates 5 min after stimulation by magnetic beads. PAG knockdown and rescue transfections as in **a**. Fold change is calculated relative to anti-CD3 stimulation (**d**) or unstimulated (**e**). Individual data points shown with median indicated of three independent experiments. *p < 0.05; **p < 0.01; ***p < 0.001; ****p < 0.0001; ns not significant.

We moved in to the Jurkat T cell line in order to incorporate the reintroduction of PAG and PAG phosphodeficient mutants (Supplementary Fig. 3b). We found that in Jurkat T cells, PAG depletion again prevented the PDL2 induced inhibition of IL-2 secretion (Fig. 3a and Supplementary Fig. 6a), as was observed in primary human T cells.

To further establish the necessity for PAG in this signaling pathway and to understand the role of the phosphotyrosines from our phospho analysis, we reintroduced wild type PAG or three individual phosphodeficient versions of PAG (Y181A, Y317A, and Y417A) and determined that wild type PAG expression rescued IL-2 inhibition (Fig. 3a and Supplementary Fig. 6a). Additionally, the phosphorylation of tyrosine 317 is necessary for this inhibition, with some contribution from phosphotyrosine 181. We did not observe any contribution from phosphotyrosine 417 (Fig. 3a and Supplementary Fig. 6a).

Increased T cell adhesion is another important functional consequence of T cell activation by anti-CD3 stimulation, which is inhibited by the addition of PDL2 in wild type Jurkat T cells^20^ (Fig. 3b and Supplementary Fig. 6b). When PAG expression is diminished there is no inhibition of T cell adhesion observed following anti-CD3 and PDL2 combined treatment (Fig. 3b and Supplementary Fig. 6b). We additionally determined that Y317 and to a lesser extent Y181 are necessary for PD-1-PAG inhibition of T cell adhesion (Fig. 3b and Supplementary Fig. 6b).

Following anti-CD3 stimulation Jurkat T cells increase surface expression of CD69 and the introduction of PDL2 inhibits this (Fig. 3c and Supplementary Fig. 6c). The depletion of PAG in these cells caused increase in CD69 surface expression despite the presence of PDL2 during stimulation (Fig. 3c and Supplementary Fig. 6c). While re-introduction of wild type PAG rescued the inhibition of CD69 expression, the 3 phosphodeficient mutants were unable to rescue (Fig. 3c and Supplementary Fig. 6c). We show in Jurkat cells that ERK phosphorylation is increased following anti-CD3 stimulation, and inhibition by PDL2 is dependent on PAG expression (Fig. 3d and Supplementary Figs. 4d and 6d). However, in the case of ERK phosphorylation we observed that none of the phosphotyrosines were solely essential in mediating this inhibition (Fig. 3d and Supplementary Figs. 4d and 6d).

Dephosphorylation of tyrosine 527 following stimulation of Jurkat cells with anti-CD3 (Fig. 3e and Supplementary Figs. 4e and 6e) increases SRC kinase activity. Phosphorylation of tyrosine 527 is high with the introduction of PDL2 in Jurkat T cells, but is diminished in PAG depleted cells (Fig. 3e and Supplementary Figs. 4e and 6e). The reintroduction of wild type PAG rescues phosphorylation (Fig. 3e and Supplementary Figs. 4e and 6e) confirming a role for PAG in this function of PD-1. Additionally, we observed some contribution from phosphotyrosine 317 as the reintroduction of this phosphodeficient mutant resulted in the least increase in phosphorylation of SRC 527. Collectively, the three tyrosines phosphorylated following PD-1 ligation contribute differentially to downstream inhibitory functions (Supplementary Fig. 6j; For all source data see Supplementary Tables 1–4).

### PAG co-localizes with PD-1 at the immunological synapse

**Fig. 4:**
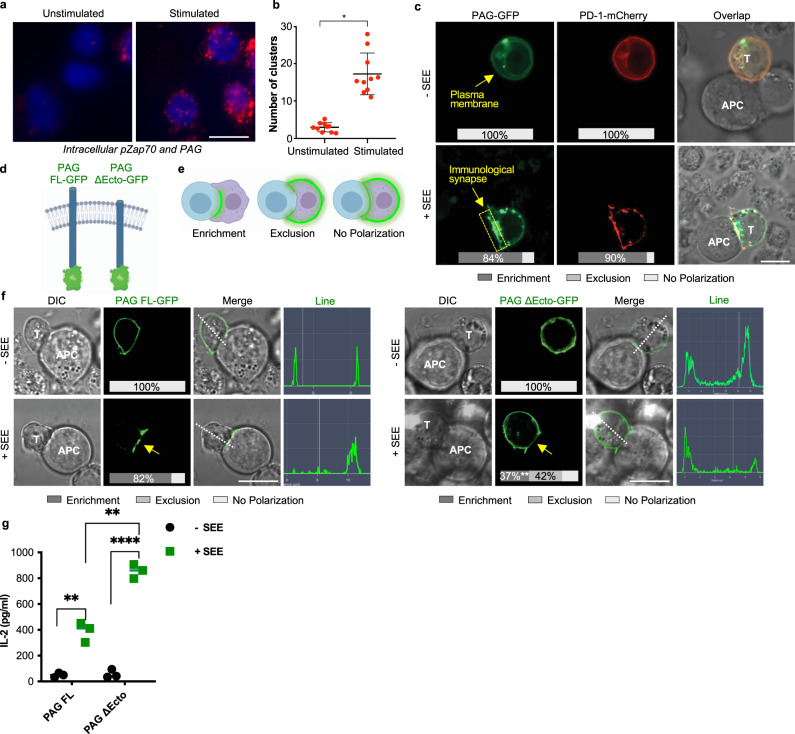
PAG colocalizes with PD-1 at the immunological synapse. **a** Jurkat T cells were stimulated with anti-CD3 and microcluster localization of phosphorylated Zap70 and PAG was assessed by the proximity ligation assay. **b** Quantification of clusters from the proximity ligation assay (**a**). **c** Jurkat cells transfected with PAG-GFP and PD-1-mCherry then incubated with Raji cells in the presence or absence of superantigen SEE. Inset percentages represent the percentage of counted cells with the represented phenotype. Fifty cells were counted for each condition in three independent experiments. **d** Schematic of PAG constructs—full length, wild type PAG (PAG FL); extracellular domain deletion of PAG (PAG ΔEcto). **e** Schematic of the characterization of GFP localization. **f** Jurkat cells transfected with PAG FL-GFP or PAG ΔEcto-GFP then incubated with Raji cells in the presence or absence of superantigen SEE. Fifty cells were counted for each condition in three independent experiments. Inset percentages represent the percentage of counted cells with the represented phenotype, statistical comparison of % enrichment between PAG FL and PAG ΔEcto is shown. Dotted white line and histogram show representative line analysis. **g** ELISA of secreted IL-2 in the supernatant of Jurkat cells transfected with PAG FL or PAG ΔEcto. Jurkat cells were co-cultured with Raji cells in the presence or absence of superantigen SEE for 24 h prior to supernatant collection. Individual data points shown with median indicated of three independent experiments; scale bars represent 10 µm. **p < 0.01; ****p < 0.0001.

At the interface of contact between a T cell and an antigen presenting cell, the immune synapse, there is a precise organization of molecules that is a critical aspect of signaling. First, to identify potential co-localization of PAG and the T cell receptor (TCR) we utilized the proximity ligation assay^21^, which allows for the visualization and quantification of protein-protein proximity <40 nm. Phosphorylated ZAP70 has been previously shown to localize in ligand-induced microclusters with the TCR^22^. We used this known localization of phosphorylated ZAP70 to establish the location of the TCR and these ligand-induced microclusters in our system. We found that phosphorylated ZAP70 and PAG co-localize following stimulation with anti-CD3 antibody (Fig. 4a, b), indicating that PAG is localized in ligand-induced micro-clusters in the immune synapse. Next, to study the localization of PD-1 and PAG in the immune synapse we utilized a co-culture of Raji B cells and Jurkat T cells. We observed that PAG and PD-1 are expressed at the plasma membrane, and upon stimulation the two proteins co-localize and are enriched at the immune synapse, with clearance from the surrounding membrane (Fig. 4c).

The short extracellular domain of PAG has an unknown contribution, if any, to PAG localization and signaling. We generated a mutant PAG that lacks this extracellular domain (PAG ΔEcto; Fig. 4d), and studied the localization of the two versions relative to the immune synapse (Fig. 4e). We found that recruitment of PAG ΔEcto to the immune synapse occurs in a significantly lower proportion of cells compared to PAG-FL (Fig. 4f). Strikingly, PAG ΔEcto remains localized to the adjacent membrane and clears or is excluded from the immune synapse upon activation (Fig. 4f). We additionally determined that PAG ΔEcto more robustly increases IL-2 secretion from activated T cells compared to overexpression of full length PAG (PAG FL; Fig. 4g) suggesting that PAG localization is probably required for its inhibitor functions.

### PAG limits the immune clearance of tumors

**Fig. 5:**
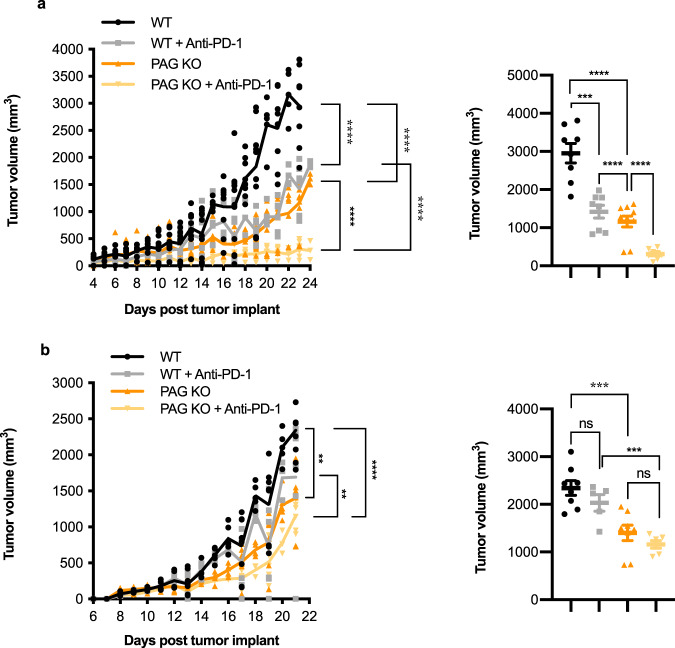
PAG is required for tumor growth. MC38 (**a**) or B16 (**b**) tumor cells were injected to WT or PAG KO mice. On day 5, 8, and 11 anti-PD-1 Abs (200 µg) were administered. Daily tumor growth as calculated from measurement by digital caliper. MC38 8–12 mice per group. B16 6–8 mice per group. **aii, bii**. Tumor volume on day 23 and day 21 of post-tumor implant, respectively, for mice that survived until the final day of the study. *p < 0.05; **p < 0.01; ***p < 0.001; ****p < 0.0001; ns not significant.

As an inhibitory signaling molecule in T cells, PAG may limit T cell activation in the context of tumors thereby influencing tumor growth. To test this, we implanted the murine colon adenocarcinoma cell line MC38 into wild type (WT) or PAG knockout (PAG KO) mice^23^ and monitored tumor growth daily. We found that PAG KO mice had limited tumor growth as compared to WT mice (Fig. 5a). MC38 tumors are responsive to anti-PD-1 administration, resulting in smaller tumors. We found that treatment of WT mice with anti-PD-1 limited tumor growth to an extent comparable to PAG KO, and of particular interest, administration of anti-PD-1 to PAG KO mice resulted in a near absence of tumors (Fig. 5a). B16 murine melanoma tumors are unresponsive to anti-PD-1 administration, and as expected we found that treatment with anti-PD-1 did not significantly inhibit tumor growth in WT mice. B16 tumor growth was hindered in the PAG KO mice, and the administration of anti-PD-1 to PAG KO mice resulted in further inhibition (Fig. 5b) suggesting the deletion of PAG sensitizes the tumors to PD-1 blockade (Tumor volumes are included in Supplementary Tables 5 and 6).

**Fig. 6:**
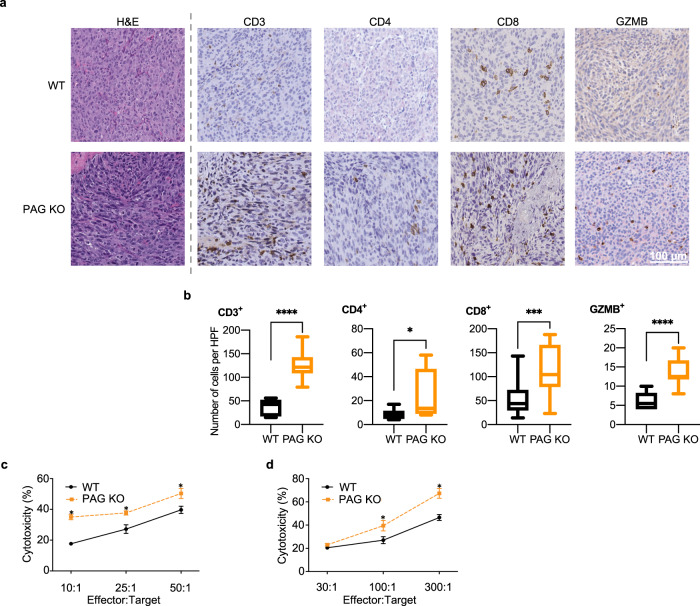
In the absence of PAG, T cells are more active. **a** Immunohistochemical staining for CD3^+^, CD4^+^, CD8^+^, and granzyme B^+^ (GZMB) infiltrating cells in MC38 tumors from WT and PAG KO mice at day 19 after tumor implantation. **b** Quantification of CD3^+^, CD4^+^, CD8^+^, and GZMB^+^ cells per high powered field (HPF) of the staining represented in **a**. Ten to fifteen HPFs were analyzed from each group. **c** IL-2 primed splenocytes (effector cells) from WT and PAG KO mice were co-cultured overnight with L929 cells (target cells). The release of LDH from the lysed target cells was measured and cytotoxicity was calculated relative to target cells cultured in the absence of primed T cells. **d** Purified CD8^+^ T cells (effector cells) were isolated from WT and PAG KO mice and co-cultured with Raji cells (target cells) pre-loaded with SEE. The release of LDH from the lysed target cells was measured and % cytotoxicity was calculated relative to target cells cultured in the absence of primed T cells. *p < 0.05; ***p < 0.001; ****p < 0.0001.

In human T cells, PAG is highly expressed in the effector population (Supplementary Fig. 7). To determine if PAG KO mice formed smaller tumors due to T cell infiltration and activation we isolated tumors to analyze for T cell presence. Within tumors isolated from PAG KO mice we observed an increased presence of CD3^+^, CD4^+^, and CD8^+^ cells (Fig. 6a, b).

**Fig. 7:**
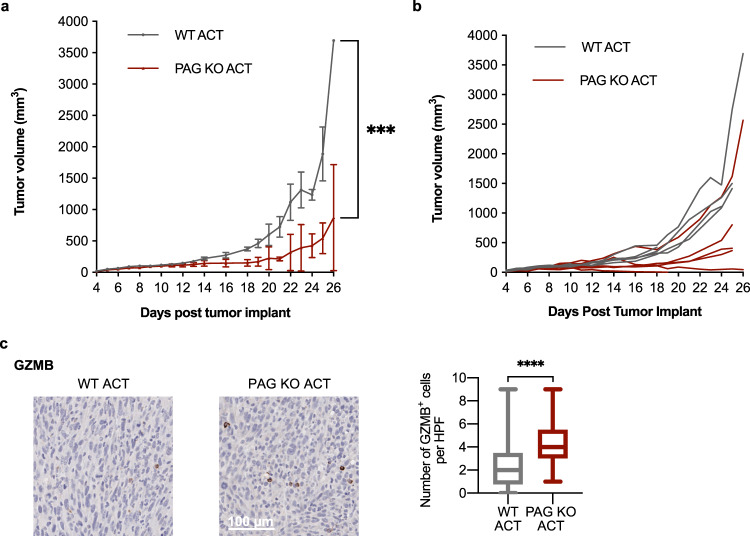
PAG depletion in T cells is sufficient for an anti-tumor effect. MC38 tumors were implanted in WT mice that received two adoptive transfers of 8.5 × 10^6^ WT or KO CD3^+^ T cells (six mice per group). **a**, **b** Daily tumor growth as calculated from measurement by digital caliper. Mean ± SEM (**a**); individual mice (**b**). **c** Immunohistochemical and quantification for granzyme B^+^ (GZMB) infiltrating cells in MC38 tumors from adoptive transfer recipient mice at day 26 after tumor implantation. ***p < 0.001; ****p < 0.0001.

To link the observed increase in tumor infiltrating T cells with effector T cell function we stained tumors for granzyme B and show increased expression of granzyme B in PAG KO tumors (Fig. 6a, b). Ex vivo, we assayed natural killer and CD8^+^ T cell cytotoxicity. We found that both natural killer and T cells isolated from PAG KO mice exhibited increased cytotoxicity (Fig. 6c, d). This leaves open the possibility that enhanced cytotoxicity in the absence of PAG contributes to the mechanism of anti-tumor response in PAG KO mice, that greatly reduced tumor growth. To further attribute the limited tumor growth in the absence of PAG more directly to T cells, we used an adoptive cell transfer of WT or PAG KO T cells into MC38 tumor bearing WT mice. We found that transfer of PAG KO T cells alone was sufficient to limit tumor growth (Fig. 7a, b). Tumor volumes are included in Supplementary Table 7. We stained tumors from adoptive transfer recipient mice for granzyme B and show increased expression of granzyme B in tumors from mice that received PAG KO CD3^+^ cells (Fig. 7c).

## Discussion

PAG is strongly associated with human cancers. A recent study reported that PAG expression quantitative trait loci (eQTL) strongly correlated with survival and response to treatment of patients with melanoma^18^. PAG protein expression level has been shown to be an unfavorable prognostic marker in patients with colon adenocarcinoma, renal cell, melanoma, acute myeloid leukemia, invasive breast, cervical squamous, and testicular cancers. PAG is also highly expressed in lymphoid malignancies^24^ and genome wide association studies identified correlation between PAG and ovarian cancer risk, survival, and response to cisplatin and paclitaxel^25^. PAG translocation (t8:8)(q21:q24) (EIF3H/PAG) was identified in several solid malignancies and among PAG variants^25,26^, rs5003154^27^ was strongly associated with severe prognosis in bladder cancer.

**Fig. 8:**
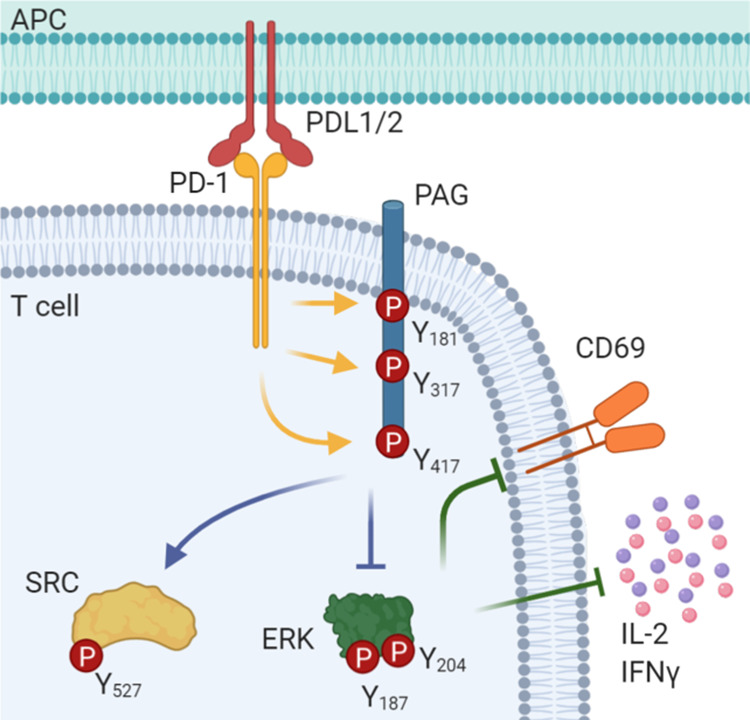
PAG is a mediator of PD-1 signaling. A model of the role of PAG in PD-1 signaling. As T cells are stimulated through the T cell receptor and PD-1 is bound to PDL1 or PDL2 PAG is phosphorylated at Y181, Y317, and Y417 and plays a necessary role in the inhibition of T cell function.

One of the proteins that was phosphorylated downstream of PD-1 was PAG, a protein that is known to inhibit multiple T cell functions (Fig. 8). While there is considerable data about the signaling pathways downstream of PAG, little is known about the functions of PAG in the context of the immune response to tumors. Our data serve not just to elucidate the mechanisms by which PAG facilitates PD-1 signals, but also suggest that the observed spotty effectiveness of anti-PD-1 in the clinic may be mediated, at least in part, through PAG signaling.

PAG KO mice have been previously shown to have no apparent defects in T cell development^23,28,29^, and an increased ex vivo response to stimulation only in T cells previously activated^28^. When PAG KO mice were studied in the experimental auto-immune encephalomyelitis (EAE) model it was found that the mice were more susceptible and that this effect was T cell intrinsic^28^. PAG is also expressed in mast cells, and has been shown to function as a positive and negative regulator of mast cell function depending on the signaling pathway involved^26^. This role for PAG as a mediator in multiple signaling cascades lends support to our observations, that PAG may also contribute to non-PD-1 mediated signaling in T cells.

Worthy of note, our study primarily focuses on signaling following PD-1 ligation by PDL2. We do not anticipate significant differences in the role of PAG downstream of signaling initiated by the two ligands (PDL1 and PDL2). We show here that there is no difference in IL-2 secretion when PAG depleted T cells are stimulated with anti-CD3 and PDL1 or PDL2 (Supplementary Fig. 4a). Additionally, our studies focus on stimulating T cells with anti-CD3 without inclusion of anti-CD28. Though studies have suggested that CD28 is the primary target for PD-1 mediated inhibition^27^, our objective in this research was to identify additional mediators of PD-1 signaling. In order to identify a role for PAG in PD-1 signaling, we employed a reductionist approach to cell stimulation relying only on the signals generated by anti-CD3 antibody and the PD-1 ligand. As is evident in our results, PD-1 does have an inhibitory effect on numerous T cell functions even in the absence of direct CD28 stimulation^30^.

There are some limitations to our studies. In the MC38 tumor model, PAG depletion appears to synergize with PD-1 blockade to impede tumor growth, suggesting that these two pathways may be non-redundant. There are several models to explain synergistic signaling as a means of enhancing response to a graded ligand; PD-1 and PAG may signal in parallel through non-redundant pathways and interact at the level of a downstream component. Alternatively, both PD-1 and PAG may be present in the same receptor complex, where PD-1 acts directly on PAG to recruit other proteins to the same complex. This model has been implicated for receptors such as TGFβ, SAX, and TKV^31,32^. It is also possible that PAG mediates PD-1 independent signaling pathways.

Overall, this study established PAG as a mediator of PD-1 signaling and function. Ligation of PD-1 with PDL2 results in inhibition of numerous T cell functions including IL-2 and IFNγ secretion, cell adhesion, ERK phosphorylation, and CD69 surface expression. We have shown that the inhibition of each of these processes is dependent on PAG, and have additionally established that the phosphorylation status of PAG downstream of PD-1 contributes to these functions (Fig. 6). The contribution of PAG to limiting tumor growth in MC38 and B16 mouse models, and enhanced cytotoxic potential might have potential translational implications.

## Methods

### LC-MS/MS analysis

Sample preparation for mass spectrometry, TMT labeling, phosphopeptide enrichment, and LC-MS/MS analysis were done as we have previously described^30^.

### General reagents

Roswell park memorial institute (RPMI) 1640 medium, Dulbecco’s modified Eagle’s medium (DMEM), Dulbecco’s phosphate-buffered saline (DPBS), and fetal bovine serum (FBS) were purchased from Life Technologies. Ficoll-Paque was purchased from GE.

### Cell culture, transfection, and stimulation

Primary T cell were isolated from unidentified donors through New York Blood Center. Consenting is not applicable. Isolation was done using RosetteSep (StemCell) followed by Ficoll-Paque. The cells were maintained in enriched media (Hepes 25 mM, sodium pyruvate 100 mM, 1% nonessential amino acids, and 1% l-glutamine) at 5% CO_2_ and at 37 °C. Primary murine splenocytes were isolated by mechanical disruption of spleens from 10 to 12 week-old mice to generate a single cell suspension. Primary murine CD8^+^ T cells were isolated from spleens of 10–12 week-old mice by CD8^+^ negative selection (StemCell). Jurkat T cells were obtained from the ATCC and maintained in RPMI medium supplemented with 10% FBS and 1% Pen/Strep (10,000 U/ml stock). Constructs were introduced into the cells by nucleofection (Lonza), efficiency of 50–70%. Cells were stimulated at a 1:3 ratio with magnetic beads conjugated with anti-CD3 (UCHT1; R&D) and IgG1 (R&D) or with anti-CD3 and PDL2-IgG1 (R&D) or with anti-CD3 and PDL1-IgG1 (R&D)^33^. Magnetic beads (Invitrogen) were coated with anti-CD3 (25%) and PDL2-Ig or PDL1-Ig fusion protein (50%); control IgG comprised the remaining total protein.

### TCGA analysis

Pan-cancer RNAseq data was analyzed by tumor type for PAG expression in hematopoietic cells. PAG expression was normalized to Protein Tyrosine Phosphatase Receptor Type C (PTPRC) and data analysis was restricted to “enriched T cells”^34^.

### DNA constructs

peGFP-N1-PAG constructs were generated by PCR cloning and site directed mutagenesis (Agilent). pmCherry-N1-PD-1 construct was generated as previously described^33^.

### Generating stable knockdown Jurkat T cells

PAG protein expression was stably knocked-down in Jurkat T cells by RNA interference using Mission shRNA plasmids (TRCN0000123273, Sigma). Lentiviral particles were generated by transfecting HEK293T cells with pMD2G, psPAX2, and the shRNA plasmid using SuperFect (Qiagen). T cells were transduced by centrifugation and selected with puromycin.

### siRNA for knockdown in primary human T cells

SMARTpool ON-TARGETplus PAG and non-targeting control siRNA (Dharmacon) were used according to the manufacturer’s instruction.

### Cytokine ELISA

To determine the concentration of secreted IL-2 and IFNγ following stimulation, human IL-2 and IFNγ ELISA kits (BioLegend) were used according to the manufacturer protocols. Primary human and Jurkat T cells were stimulated with antibody-coated beads for 24–48 h prior to supernatant collection and analysis.

### Adhesion assay

T-cell adhesion to fibronectin-coated plates was performed as we previously described^35^. Briefly, cells were labeled with CFSE, stimulated, plated on coated wells, incubated for 15 min, and nonadherent cells removed by serial washes. The number of adherent cells remaining, expressed as a percentage of the total number of labeled cells, was determined with a fluorescent plate reader (Synergy HT, BioTek Instruments). Each experiment was performed three times.

### Immunoprecipitation and western blot

Cell lysates were mixed with anti-GFP monoclonal antibody coupled to agarose beads to enrich GFP-tagged proteins according to the manufacturer’s protocols (MBL). Pull-down lysates were separated by Tris-glycine PAGE, transferred to nitrocellulose membranes, and visualized as described^20^. The following antibodies were used for biochemical assays: anti-phosphotyrosine (4G10; Millipore), anti-GFP-agarose (D153; MBL), anti-GFP (118144600; Roche), anti-pERK (9106; Cell Signaling), anti ERK (4695; Cell Signaling), anti-pSRC (2105; Cell Signaling), anti-SRC (2108; Cell Signaling), anti-pZap70 (2701; Cell Signaling), anti-actin (1616; Santa Cruz), anti-PAG (MEM-255, Origene). Prior to pERK and pSRC analysis, primary human and Jurkat T cells were stimulated for 5 min, lysed in modified 1× RIPA buffer containing protease and phosphatase inhibitors. Lysates were separated by Tris-glycine PAGE, transferred to nitrocellulose membranes, and visualized as described^20^.

### Flow cytometry

Non-permeabilized T cells were stained with the fluorescently conjugated antibody specific for CD69 (H1.2F3; BioLegend) in FACS Buffer [HBSS without Ca^2+/^Mg^2+^, FBS (3%), NaN_3_ (0.02%), and CaCl_2_ (2.5 mM)], then washed and fixed in 1% paraformaldehyde. PAG-GFP expressing cells were fixed in 1% paraformaldehyde. Events were recorded using FACSCalibur (BD), and analyzed using FlowJo software (Ver. 10.1r7).

### Proximity ligation assay

The proximity ligation assay (PLA) kit (Sigma-Aldrich) was used according to the manufacturer protocol. Millicell 8-well glass slide plates were coated with poly-l-lysine for 1 h at 5% CO_2_ and 37 °C. Jurkat T cells were then added to the slide and allowed to adhere for 1 h. Anti-CD3 was used to stimulate the cells for 5 min at 5% CO_2_ and 37 °C. Images were acquired using a Zeiss Imager (Carl Zeiss Microimaging).

### Jurkat/Raji conjugate formation assay

Raji B cells were preloaded for 45 min at 37 °C with 1 mg/ml Staphylococcus enterotoxin E (SEE). Jurkat and Raji cells were then mixed in a 1:1 ratio and after 15 min plated on poly-l-lysine (10 µg/ml)-coated µ-dish 35 mm (ibidi). Images were acquired with an inverted Zeiss 700 laser scanning confocal microscope (Carl Zeiss Microimaging). Each experiment was performed three or more times. Forty conjugates were scored in each experiment.

### Mice and tumor cell lines

Male, 6–12-week-old C57BL/6 (B6) wild-type (WT) or PAG knock-out (PAG KO)^23^ mice were used in all animal studies. Animal studies were approved by the Columbia University Institutional Animal Care and Use Committee. The WT and PAG KO mice in all studies were littermates, assuring a homogeneous genetic background. The murine colon adenocarcinoma (MC38) colon carcinoma cells were a gift from Ben Neel of New York University. Prior to use, MC38 cells were authenticated by simple sequence length polymorphism (SSLP). The MC38 cells were maintained in DMEM supplemented with heat-inactivated fetal bovine serum (FBS; 10%) and penicillin-streptomycin (P/S; 1% 10,000 U/ml stock) and grown at 37 °C with 5% CO_2_. B16 cells were a gift from Eva Hernandez of New York University. Cells were passaged prior to storage and thawed and passaged twice prior to implantation for all described tumor experiments. All cell lines were determined to be free of mycoplasma (Lonza).

### Tumor model

MC38 (1 × 10^6^) and B16 (0.5 × 10^6^) cells were implanted subcutaneously in the right hind flank of 6–10-week-old mice. Tumor growth was monitored using electronic calipers and calculated according to the formula: *V* = length × width^2^ × 0.52. For survival experiments, mice were euthanized when tumor size reached maximum volume or when tumors became ulcerated. For adoptive cell transfer experiments, CD3^+^ cells were purified by negative selection (Mojosort, BioLegend) from age-matched WT or PAG KO splenocytes. 8.5E6 CD3^+^ cells were injected intravenously once MC38 tumors reached a measurable size and again 3–4 days later for a total of two adoptive transfer injections. For immunohistochemistry, tumors were fixed in 10% neutral buffered formalin then paraffin embedded and cut into 5 µm sections. Slices were stained with anti-CD3 (eBioscience), anti-CD4 (Abcam), anti-CD8 (Cell Signaling Technology), anti-granzyme B (Abcam), and bound antibody was detected with peroxidase-based staining.

### Cytotoxicity assays

L929 target cells—IL-2 primed splenocytes (effector cells) from WT and PAG KO mice were co-cultured overnight with L929 cells (target cells). The release of LDH from the lysed target cells was measured by the LDH cytotoxicity kit (ThermoFisher), and cytotoxicity was calculated relative to target cells cultured in the absence of primed T cells. Raji target cells—Murine splenocytes were cultured with 1 µg/ml SEE and 1000 U/ml mIL-2 (Miltenyi) for 72 h, followed by isolation of CD8^+^ T cells using isolation kit (Miltenyi). SEE loaded Raji B cells (target cells) were mixed with CD8^+^ T cells at different ratios, as indicated, and cytotoxicity was tested using LDH cytotoxicity kit (ThermoFisher).

### Statistics and reproducibility

Values are reported as means ± SEM. Statistical analyses were performed using Student’s *t*-test. For tumor growth curves a simple linear regression with statistical comparison of the slopes was performed. All statistical analyses were performed using GraphPad Prism 8.

### Reporting summary

Further information on research design is available in the Nature Research Reporting Summary linked to this article.

## Supplementary information

Supplementary Information

Reporting Summary

## Data Availability

The raw phosphoproteomic data are available at MassIVE, RRID:SCR_013665. The MS raw files are accessible under MassIVE ID: MSV000084813. Source data underlying plots shown in figures are provided in Supplementary Tables. All other data are available from the corresponding author on reasonable request.
