# Supplementary material for: Transmembrane adaptor protein PAG is a mediator of PD-1 inhibitory signaling in human T cells
Source: Commun Biol. 2021 Jun 3;4:672. doi: 10.1038/s42003-021-02225-8 (PMC8175585; doi:10.1038/s42003-021-02225-8)
Supplement: Supplementary file 1 — Supplementary Information [file 42003_2021_2225_MOESM1_ESM.pdf]

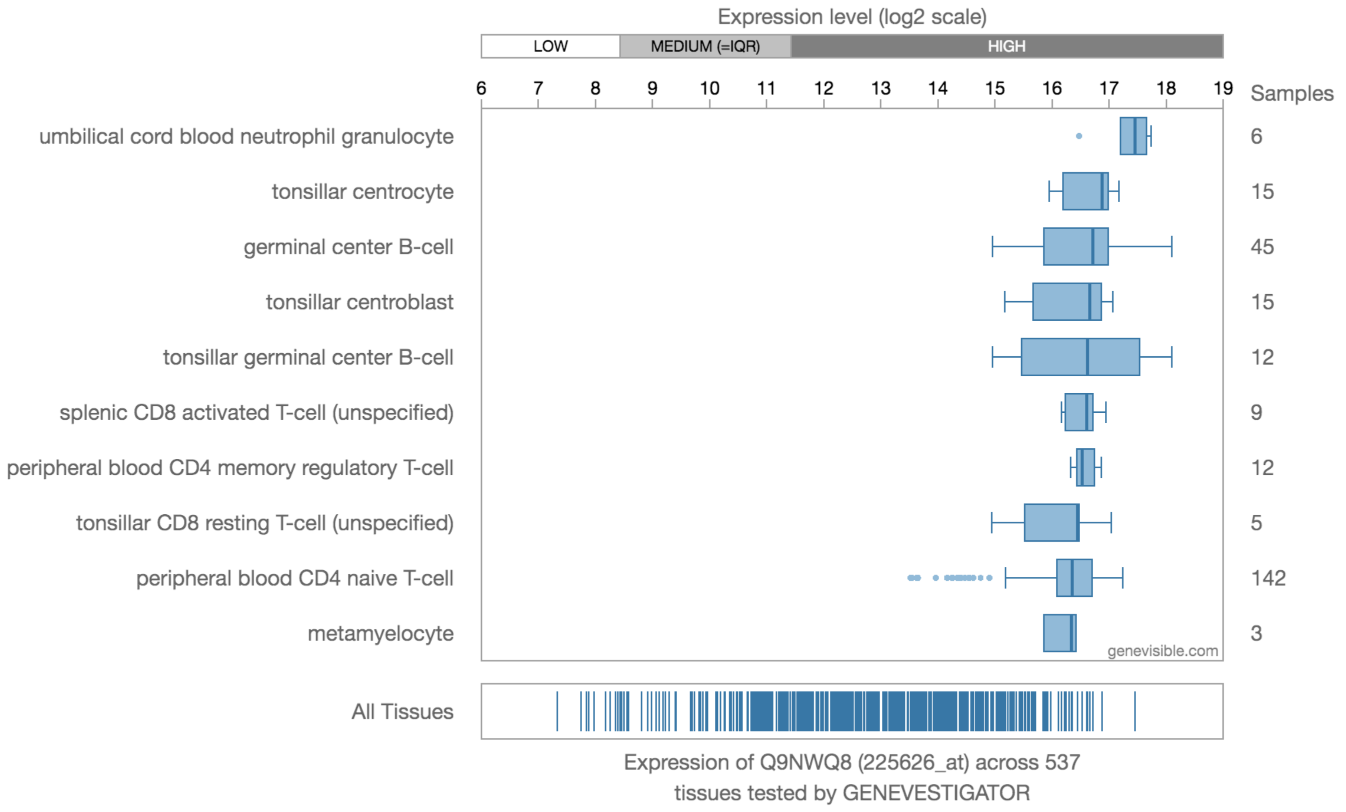

Supplementary Figure 1. PAG protein expression in the top 10 highest expressing tissues[14].

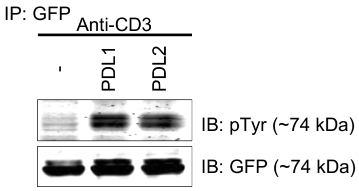

Supplementary Figure 2. Jurkat cells expressing PAG-GFP were stimulated with anti-CD3 alone or with PDL1 or PDL2. PAG-GFP was enriched by immunoprecipitation of the lysates and phosphotyrosine was detected by immunoblot. Representative blot of three independent experiments is shown.

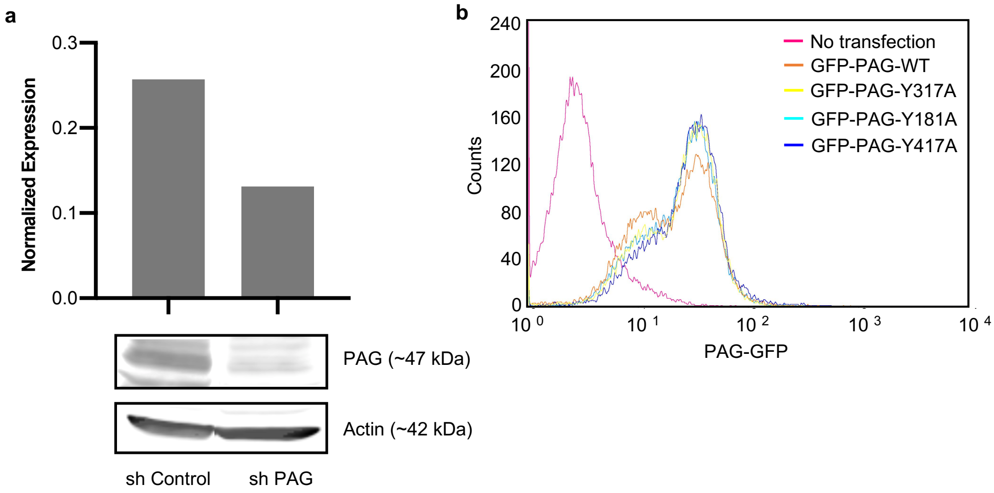

Supplementary Figure 3. a. Jurkat cells expressing either a non-targeting shRNA (shControl) or a PAG targeting shRNA (shPAG) were lysed and run on a western blot. Immunoblot shows PAG and actin protein levels. Representative blot is shown. **b.** Jurkat cells expressing sh PAG were transfected with wild type full length PAG or phosphodeficient versions and GFP expression was quantified by flow cytometry.

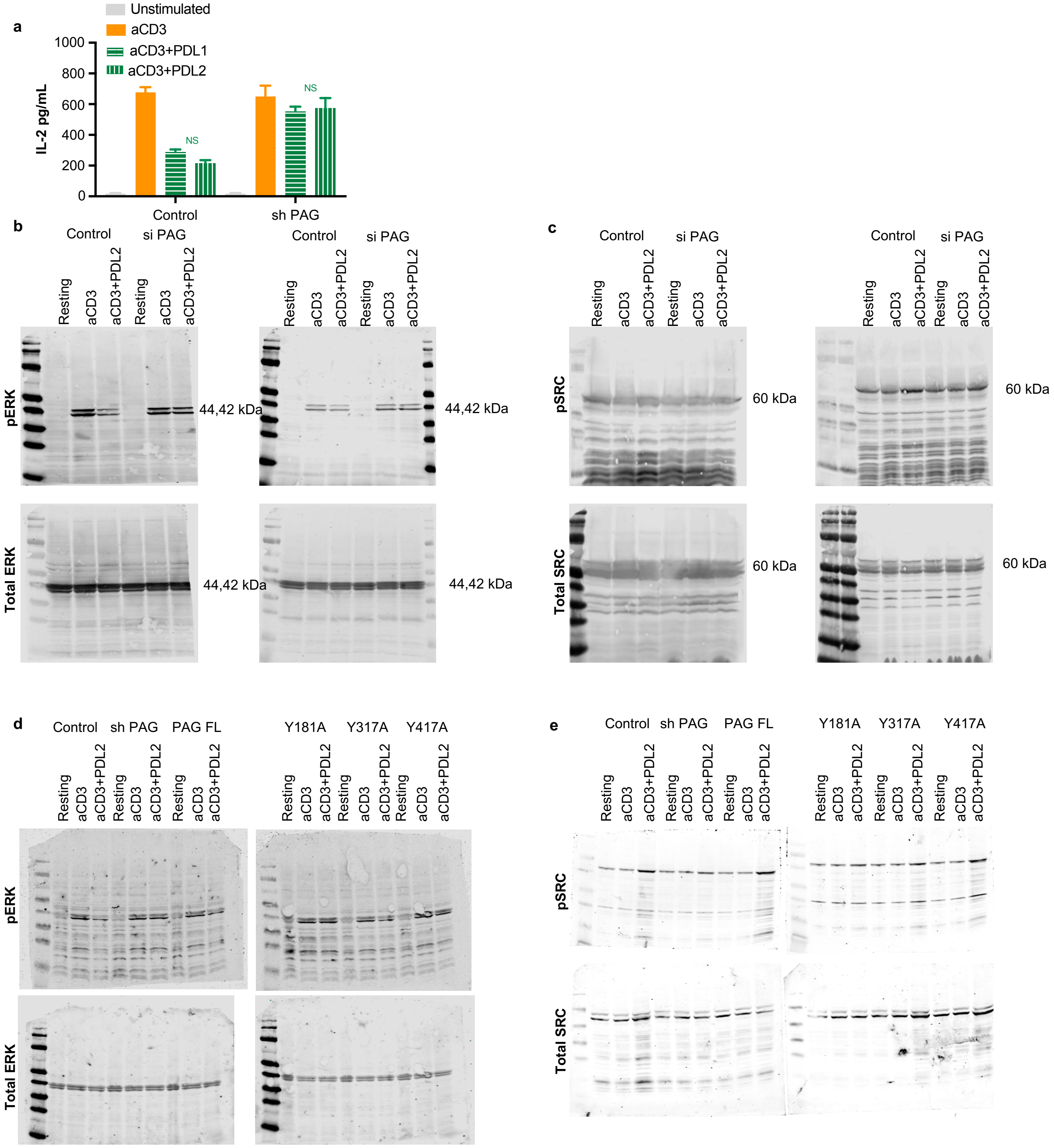

Supplementary Figure 4. a. ELISA of secreted IL-2. Jurkat cells expressing sh Control or sh PAG were stimulated for 24 hours before supernatants were collected. Bars represent mean ± SEM of three independent experiments. **b - e.** Representative western blots of primary T cells (b, c) and Jurkat cells (d, e) expressing si/sh control or si/sh PAG and rescue transfections with various versions of PAG into shPAG expressing Jurkat cells as indicated. **d.** Table summarizing the contributions of phosphotyrosines 181, 317, and 417 to the assessed T cell functions and PD-1 signaling.

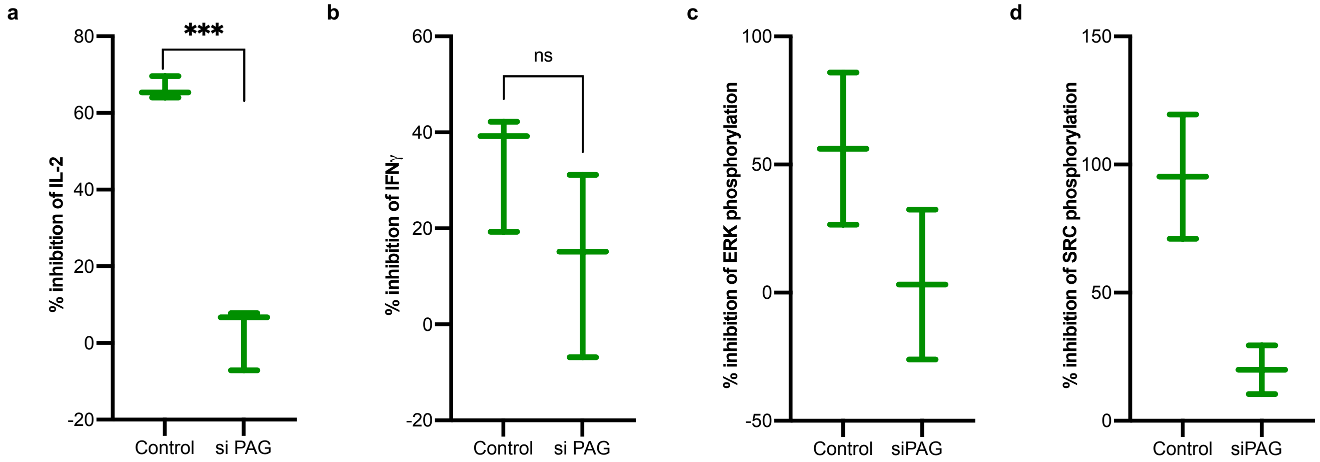

Supplementary Figure 5. a. and b. ELISA of secreted IL-2 (a) and IFNγ (b) in the supernatants of primary human $CD3^+$ T cells collected 48 hours following stimulation by magnetic beads. T cells either expressed a non-targeting siRNA (control) or PAG targeting siRNA pool (siPAG). **c and d.** Phosphorylated ERK (c) and phosphorylated SRC (d) were detected by western blot of human $CD3^+$ T cell lysates 5 minutes after stimulation by magnetic beads. Fold change is calculated relative to anti-CD3 stimulation (c) or unstimulated (d). *$p<0.05$; **$p<0.01$; ***$p<0.001$; ****$p<0.0001$; ns not significant.

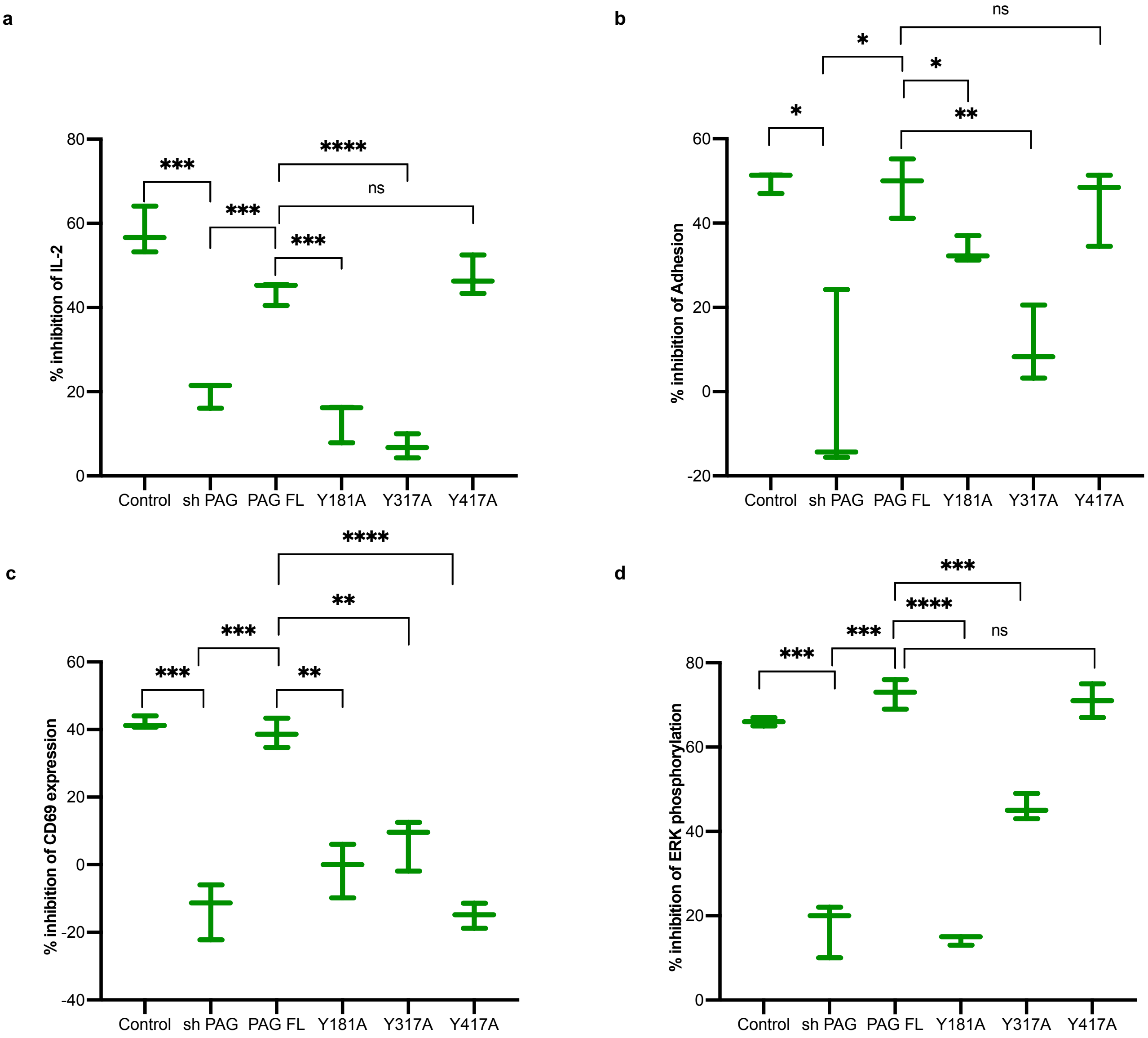

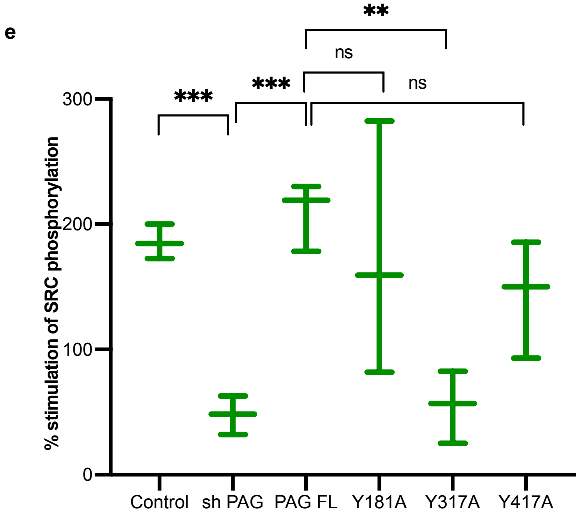

f

Assay	WT	Y181	Y317	Y417
IL-2	+	+	++	o
Adhesion	++	o	++	o
CD69	++	++	++	++
pERK	+	o	o	o
pSRC	++	+	++	o

o no contribution, **+** some contribution, **++** necessary role

Supplementary Figure 6. a. ELISA of secreted IL-2 in the supernatants of Jurkat cells collected 24 hours following stimulation by magnetic beads. Jurkat T cells either expressed a non-targeting shRNA (control) or PAG targeting shRNA (shPAG). For rescue transfections, shPAG Jurkats were transiently transfected with full length, wild type PAG (PAG FL) or with phosphodeficient mutants (Y181A, Y317A, Y417A). **b.** Adhesion assay of Jurkat cells to fibronectin following stimulation for 15 minutes. The number of adherent cells remaining, expressed as a percentage of the total number of labeled cells, was determined with a fluorescent plate reader. PAG knockdown and rescue transfections as in (A). **c.** Percentage of Jurkat cells expressing CD69 on the surface following 24 hour stimulation, measured by flow cytometry. PAG knockdown and rescue transfections as in (A). **d and e.** Phosphorylated ERK (D) and phosphorylated SRC (E) were detected by western blot of Jurkat lysates 5 minutes after stimulation by magnetic beads. PAG knockdown and rescue transfections as in (A). % inhibition is calculated by 100 – CD3+PDL2 ÷ CD3 * 100. Bars represent the mean ± SEM of three independent experiments. **f.** Table summarizing the contribution of each phosphotyrosine position to individual T cell functions assayed. *p<0.05; **p<0.01; ***p<0.001; ****p<0.0001; ns not significant.

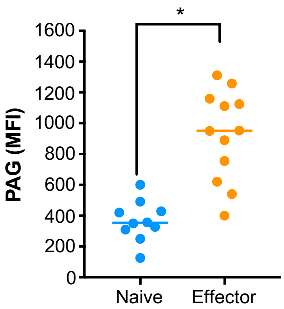

Supplementary Figure 7. Human naïve ($CD8^{+}CCR7^{+}CD62L^{+}$) and effector ($CD8^{+}CCR7^{-}CD62L^{-}$) T cells were stained with intracellular anti-PAG antibody and analyzed by flow cytometry. MFI - median fluorescent intensity.

Supplementary Table 1. IL-2 ELISA

Primary human T cells	Unstimulated			aCD3			aCD3+PDL2		
Control	20	25	0	661	640	655	201	230	227
si PAG	20	30	32	553	578	610	510	619	569

Jurkat cells	Unstimulated			aCD3			aCD3+PDL2		
Control	20	5	23	805	946	902	289	410	422
sh PAG	35	22	32	871	953	944	731	748	741
PAG FL	25	23	23	754	835	810	411	457	482
PAG Y181A	19	27	15	750	848	821	691	710	688
PAG Y317A	12	14	27	737.8349	790	780	688	756	702
PAG Y417A	33	18	29	777	810	838	440	435	398

Supplementary Table 2. IFNg ELISA

	Unstimulated			aCD3			aCD3+PDL2		
Control	27	71	46	1298	1301	1514	750	1050	920
si PAG	154	30	36	1650	1453	1404	1400	1000	1500

Supplementary Table 3. Percent inhibition of CD69 expression

	Unstimulated			aCD3			aCD3+PDL2		
Control	3	3	0	51	59	54	30	33	32
sh PAG	3	4	5	54	62	67	66	69	71
PAG FL	7	6	6	49	57	53	32	35	30
PAG Y181A	8	6	2	51	45	50	56	45	47
PAG Y317A	7	6	11	52	56	54	47	49	55
PAG Y417A	6	7	10	27	35	32	31	39	38

Supplementary Table 4. Percent inhibition of adhesion

	Unstimulated			aCD3			aCD3+PDL2		
Control	10	14	11	35	37	34	17	18	18
sh PAG	14	18	12	28	32	33	32	37	25
PAG FL	8	10	11	30	38	34	15	17	20
PAG Y181A	9	13	7	32	27	31	22	17	21
PAG Y317A	12	16	14	31	36	34	30	33	27
PAG Y417A	8	13	17	37	29	33	18	19	17

Supplementary Table 5. MC38 Tumor Measurements

Days	WT												WT + Anti-PD-1											
1	25.38	55.14	18.37	38.53	35								15.1	30.77	14.98	27.7	22							
2	71	66.17	55.57	131.92	89								29	34.82	30.77	30.77	26							
3	117	122.45	168.31	106.96	128								75	94.38	21.64	44.13	58							
4	130	142.41	217	173.97	144	0	137.795		0				77	75	73.32	63.9	78							
5	242.61	172	321	186.44	215	0	206.822		85.82				83.45	96	121.6	107.52	96							
6	385	209	283.34	166.74	230	0	263.709		192.584				46	140.4	191.94	99.72	126							
7	406	258.44	253.51	319.37	282	0	263.178		171.921	13	6	0.5	74	128	232.81	157.48	119.29	40	0	28.9				
8	394	330	327.71	288	316	308.386	285.0369	71.345	167.225				136.71	205	173	191.87	149.5							
9	406	353	396.8	402	460	498.026	320.183	62.086	239.61				187.8	198	244.46	214.97	222.11							
10	679.59	356.72	644.64	416	559	412.139	315.268	55.729	309.468	25.6	34.4	6	308.74	177	389.81	324.68	203.84	54.7	18	32				
11	706.97	360.72	728	388	576	439.206	442.512	67.7	320.023				428.69	150.92	318.42	413.27	138.53							
12	710.22	547.82	801	622	616	474.235	493.067	82.79	446.59	27.5	42	6.4	421.34	279.34	606.5	712.81	242.62	108	20.5	66.3				
13	942	642.55	904	661.74	787	1159.27	494.31	122.14	551.609				713	343.16	638.19	779	382.67							
14	1023	847	1066.42	953.24	972	948.579	552.337	140.041	489.995	49.5	63.8	13.5	801.22	331.02	859.95	1202	561.6	123	23	176.4				
15	1180.05	1075	1300.62	1134.02	1001								798	381.06	741.41	1380.87	383.21							
16	1249	1240.75	1319	1655	1361	1398.769	851.633	239.054	489.995				617	568.22	1178.04	1202.81	503.79							
17	1486.94	1476	1531	2449.41	1396	1557.991	1085.625	179.7444	1128.868	494.6	198.5	35.3	811	565.71	871.45	1221	498.39	151.9	65.8	181.5				
18	1510	1599	2205	1891	1499	1995.209	1134.7999	1432.954	1287				887	565.71	888	1229	890.56							
19	2228.79	1602	2924.93	2153.34	2226	2857.511	1464.737		1597.866	2099.2	530.6	505	1107	670.03	621	839	796.89	372.6	368.6	380.9				
20	2721	1819	2782.54	3112	2609								1238.45	832.53	770	910	761.3							
21	2811	2115.94	3321.19	3339	2896					2615.5	1503.6	1687.5	1309	1115.38	894	1103	1097.46	625	539.1	585				
22	2985	2529	3544	3620	3160								1877	1510	1871	1671	1529							
23	3296	2575	3717	3812	3319					2924.7	1813	2176	1977	1560	1799	1790	1617	925.8	864	828.2				
24														1819		1937	1870.21							

Days	PAG KO												PAG KO + Anti-PD-1											
1	22.36	47.39	17.93	28.3	28								15	35.89	28	34.37	18.72							
2	132.05	76.15	11.4	61.18	70								21	53.35	18.65	61.18	64.13							
3	138.03	81.71	6.97	78.45	75								27	54.64	18	40.77	75.94							
4	198.6	114.54	222	121.4	91	0	0	35.8	0				29	63.79	48.19	74.28	79.98							
5	242.05	309.24	313	90.13	93	39.076	104.956	39.9	28.925				42	118.15	39.72	50.62	125.23							
6	231.08	268	621.1	171.37	179	29.575	204.462	79.681	119.5168				43	125	42.61	136.44	196.61							
7	331.14	316.16	487.28	201.29	263	78.363	433.102	100.786		0	48.6	0	39	135.51	58.59	158.18	154.52	0	0	6				
8	227.23	391	653.02	229.13	351	129.23	179.61	66.21	198.08256				45	87.72	80.11	121.9	182.68							
9	256.2	342.81	540.04	160.63	389	61.575	290.368	120.956	187.43296				52	57.7	78.29	55.59	198.16							
10	157.77	369.5	712.36	314.68	387	117.807	286.026	118.521		22.1	90	62.5	49	87.82	93.08	137.92	328.6	0.6	6.6	7.5				
11	418.8	462	817	157.09	532	136.628	286.807	103.151					53	157.39	162.38	161.07	227.27							
12	325.62	668.03	472	424.15	471	130.024	307.683	136.448	224.24688	24	94.6	83.1	47	111.65	222.86	62.92	108	1	7.3	30.4				
13	473.05	474.27	397	246.63	399	135.266	362.054	138.476	222.1326016				41	184.86	278.83	122.45	115							
14	435.73	1069.22	595	281.5	595	166.972	451.149	169.296	344.8345285	94.6	130.2	87.5	29	97.5	264.49	96.21	120	6.6	18.9	32				
15	519.47	920.88	487	292.08	554				415.35936				47	246.63	282.53	119.86	172							
16	602.49	491	490	245.8	485	166.972	451.149	169.296	499.50992				52	212.84	472	193.98	209							
17	447.79	461	521	595.11	601	180.031	732.321	245.7	467.11808	189.2	224.3	118.8	61	249.77	393	240.48	225	166.6	40	56.4				
18	447.79	497	559	595.11	537	223.911	676.829	205.705	511.0716				60	261	298	241	227							
19	951.68	509	851	730.34	648	186.235	881.776	311.413	717.1008	474	355.2	500	64	251	359	246	229	237.2	137.7	245				
20	1200	666	1013	898.6	801	217.512	907.473	180.279	981.28836				98	257	370	336	264							
21	1289	893	1106	898.9	989	288.343		233.955	1115.48164	1054.2	1239.8	1098.5	81	218	308	252	212	352	215.6	320				
22	1448	921	1307	1239.11	1197	339.381		350.664					101	239	330	207	207							
23	1619	1071	1510	1407	1278	374.545		358.337		1259.1	1540.8	1183	96	340	362	280	172	496.9	267.2	445.5				
24	1698		1612	1503									107	458	280									

Supplementary Table 6. B16F10 Tumor Measurements

Days	WT								WT + Anti-PD-1							
1	0								0							
2	0								0							
3	0								0							
4	0								0							
5	0								0							
6	0								0							
7	0								0							
8	62.5	72	108	75	43				42	63	51					
9	85	100	100	126	97				107	126	97					
10	110	133	110	140	167				150	109	128					
11	135	126	143	270	236				203	155	175					
12	158.4375	155	169	408	386.334				239.0625	288	340					
13	251	178	207	455	420	61.3	48.4	48.8	328	352	397	67.6	56	0		
14	354	301	360	573	298				269	494	449					
15	465.75	710	698	606	564.9188				524	627	521					
16	769	993	1097	725	615				624	757	674					
17	1102	1205	1108	847	799	171.5	162	486	796	827	973	288	196	0		
18	1547	1403	1650	1350	1220				1187	1080	1280					
19	1688	1620	2017	1505	1521	744.2	635	726	1071	1231	1533	299.2	282.9	0		
20	1801	2017	2219	2403	2101				1371	1775	1895					
21	3105	2258	2730	2437	2450	1890	1793.4	2073.6	1852	2280	2364	1428.8	2229.2	0		

Days	PAG KO								PAG KO + Anti-PD-1							
1	0								0							
2	0								0							
3	0								0							
4	0								0							
5	0								0							
6	0								0							
7	0								0							
8	154.791	125	117	108	79				41	37	66					
9	160	140	170	127	64				56	56	75					
10	165	149	199	150	76				73	112	101					
11	170	149	213	176	99				90	179	144					
12	179.5625	91	218	253.6285	110.808				141	250	204					
13	202	155	287	289	186	15.4	76.3	35.3	172	246.63	119.86	30.4	27.2	19.2		
14	240	206	310	310	265				229	212.84	193.98					
15	288	179.5625	378.895	324	313.8266				270	249.77	250					
16	351	303	420	441	501				274	269	289					
17	527	450	556	600	681	64	484.3	766.1	180	301	321	395.1	423.2	115		
18	590	636	753	633	789				455	447	305					
19	750	842	831	1046	1190	136.1	465.8	1023.4	603	460	337	885.6	617.2	180		
20	1090	1210	1256	1330	1644				762	952	631					
21	1860	1407	1548	1472	1950	727.4	738.1	1502.2	950	1308	903	1381.8	1264.8	1140.8		

Supplementary Table 7. Adoptive Cell Transfer Tumor Measurements

Days	WT ACT						PAG KO ACT					
4	15.110016	12.025874	11.2168125	36.1	33.7211875	24.4089375	6.593535	11.2519425	7.10124	24.5934315	33.9015555	17.4421875
5	47.82528	38.406175	37.866318	59.5	70.2	43.5	39.9906315	25.80565	51.5780485	46.6	48.6	27.5
6	83.291475	67.268268	67.528027	62.7	72.498168	40.490464	69.679106	37.9516025	67.537072	56.3022495	44.976384	22.775877
7	108.409291	96.882339	91.0940625	79.4	85.86368	86.3374	65.28918	55.4617125	75.750552	69.049096	81.832842	55.581058
8	117.855744	110.230116	95.84685	92.7	83.4967255	113.872676	69.917328	49.132252	69.727686	96.1253835	94.7000925	49.515176
9	112.484714	93.6096	93.9168625	112	97.314392	95.50135	70.909952	43.52175	75.1078125	130.112864	149.3284	86.5628
10	133.3223	96.86911	105.456	148.2	92.31453	130.447284	67.162368	70.153864	68.8286255	146.849472	157.074515	84.21745
11	131.500567	123.1254	132.631448		124.436316	120.115331	75.146566	98.464382	92.530107	146.202	203.738328	38.263752
12	176.112792	112.749325	158.129885		118.5921	158.042836	78.381056	92.942154	81.238113	198.724314	180.895478	44.255232
13	194.490432	137.5528	175.950425		162.1	202.6	89.010492	107.516448	98.069562	238.9	179.7	23.8
14	206.525344	164.122378	206.3484		231.6	301.2	96.0362325	97.185816	79.27596	311.2	252.8	22.1
15												
16	232.084845	186.024192	264.6		241.4	445	93.5712	99.790894	112.402404	436.1	132.5	9
18	353.773036	304.760475	421.673548		322.6	458.8	108.684834	150.669018	145.023232	382.2	110.9	4.2
19	491.306634	393.663888			325.5	631.6	142.93073	155.79648	164.60352	495.5	60.3	0
20					424.7	768.5				589.1	86.4	0
21	887.48784	559.651968					181.467841	187.965603	270.678112			
22					827.4	1404				894.7	54.3	0
23					1025.5	1597.4				1136.6	40.8	0
24	1261.70563	1086.89319			1112.8	1472.2	391.039101	298.415513	537.410336	1273	49.1	0
25	1500.63188	1412.61611				2745.3	405.065026	365.523463	802.102928	1616.4	58.5	0
26						3693.5				2565.6	45.9	0
27												
28												
