# Supplementary material for: Transmembrane adaptor protein PAG is a mediator of PD-1 inhibitory signaling in human T cells
Source: Commun Biol. 2021 Jun 3;4:672. doi: 10.1038/s42003-021-02225-8 (PMC8175585; doi:10.1038/s42003-021-02225-8)
Supplement: Supplementary file 2 — Reporting Summary [file 42003_2021_2225_MOESM2_ESM.pdf]

nature research

Corresponding author(s): Ada Mor

Last updated by author(s): Mar 24, 2021

Reporting Summary

Nature Research wishes to improve the reproducibility of the work that we publish. This form provides structure for consistency and transparency in reporting. For further information on Nature Research policies, see our Editorial Policies and the Editorial Policy Checklist.

Statistics

For all statistical analyses, confirm that the following items are present in the figure legend, table legend, main text, or Methods section.

n/a	Confirmed	
☐	☒	The exact sample size (n) for each experimental group/condition, given as a discrete number and unit of measurement
☐	☒	A statement on whether measurements were taken from distinct samples or whether the same sample was measured repeatedly
☐	☒	The statistical test(s) used AND whether they are one- or two-sided<br>*Only common tests should be described solely by name; describe more complex techniques in the Methods section.*
☐	☒	A description of all covariates tested
☐	☒	A description of any assumptions or corrections, such as tests of normality and adjustment for multiple comparisons
☐	☒	A full description of the statistical parameters including central tendency (e.g. means) or other basic estimates (e.g. regression coefficient) AND variation (e.g. standard deviation) or associated estimates of uncertainty (e.g. confidence intervals)
☐	☒	For null hypothesis testing, the test statistic (e.g. F, t, r) with confidence intervals, effect sizes, degrees of freedom and P value noted<br>*Give P values as exact values whenever suitable.*
☐	☒	For Bayesian analysis, information on the choice of priors and Markov chain Monte Carlo settings
☐	☒	For hierarchical and complex designs, identification of the appropriate level for tests and full reporting of outcomes
☐	☒	Estimates of effect sizes (e.g. Cohen's d, Pearson's r), indicating how they were calculated

Our web collection on statistics for biologists contains articles on many of the points above.

Software and code

Policy information about availability of computer code

Data collection	NA
Data analysis	NA

For manuscripts utilizing custom algorithms or software that are central to the research but not yet described in published literature, software must be made available to editors and reviewers. We strongly encourage code deposition in a community repository (e.g. GitHub). See the Nature Research guidelines for submitting code & software for further information.

Data

Policy information about availability of data

All manuscripts must include a data availability statement. This statement should provide the following information, where applicable:

- Accession codes, unique identifiers, or web links for publicly available datasets
- A list of figures that have associated raw data
- A description of any restrictions on data availability

The raw phosphoproteomic data are available at MassIVE, RRID:SCR_013665. The MS raw files are accessible under MassIVE ID: MSV000084813. All other data are available from the corresponding author on reasonable request.

Field-specific reporting

Life sciences study design

All studies must disclose on these points even when the disclosure is negative.

Sample size	Assays were repeated a minimum of 2 independent experiments with technical replicates, statistical analysis was done on independent experiments not technical replicates. A minimum of 3 mice were included in each group.
Data exclusions	No data exclusion.
Replication	All attempts at replication were successful.
Randomization	Allocation of cells and animals into experimental groups was random.
Blinding	Investigators were not blinded in this study.

Reporting for specific materials, systems and methods

We require information from authors about some types of materials, experimental systems and methods used in many studies. Here, indicate whether each material, system or method listed is relevant to your study. If you are not sure if a list item applies to your research, read the appropriate section before selecting a response.

Materials & experimental systems

n/a	Involved in the study	
☐	☒	Antibodies
☐	☒	Eukaryotic cell lines
☒	☐	Palaeontology and archaeology
☐	☒	Animals and other organisms
☒	☐	Human research participants
☒	☐	Clinical data
☒	☐	Dual use research of concern

Methods

n/a	Involved in the study	
☒	☐	ChIP-seq
☒	☐	Flow cytometry
☒	☐	MRI-based neuroimaging

Antibodies

Antibodies used	The following antibodies were used for biochemical assays: anti-phosphotyrosine (4G10; Millipore), anti-GFP-agarose (D153; MBL), anti-GFP (118144600; Roche), anti-pERK (9106; Cell Signaling), anti ERK (4695; Cell Signaling), anti-pSRC (2105; Cell Signaling), anti-SRC (2108; Cell Signaling), anti-pZap70 (2701; Cell Signaling), anti-actin (1616; Santa Cruz), anti-PAG (MEM-255, Origene). with anti-CD3 (UCHT1; R&D) for functional stimulation. Slices were stained with anti-CD3 (eBioscience), anti-CD4 (Abcam), anti-CD8 (Cell Signaling Technology), anti-granzyme B (Abcam).
Validation	Appropriate controls were included in the study to validate the antibodies.

Eukaryotic cell lines

Policy information about cell lines

Cell line source(s)	The murine colon adenocarcinoma (MC38) colon carcinoma cells were a gift from Ben Neel of New York University. B16 cells were a gift from Eva Hernandez of New York University. Jurkat cells were purchased from ATCC.
Authentication	Prior to use, MC38 cells were authenticated by simple sequence length polymorphism (SSLP). B16F10 and Jurkat cell lines were not validated
Mycoplasma contamination	All cell lines are tested regularly by Lonza mycoplasma detection kit.
Commonly misidentified lines (See ICLAC register)	NA

Animals and other organisms

Policy information about studies involving animals; ARRIVE guidelines recommended for reporting animal research

Laboratory animals	Male, 6-12-week-old C57BL/6 (B6) wild-type (WT) or PAG knock-out (PAG KO)23 mice were used in all animal studies. Animal studies were approved by the Columbia University Institutional Animal Care and Use Committee. The WT and PAG KO mice in all studies were littermates, assuring a homogeneous genetic background.
Wild animals	NA

Field-collected samples	NA
Ethics oversight	nimal studies were approved by the Columbia University Institutional Animal Care and Use Committee.

Note that full information on the approval of the study protocol must also be provided in the manuscript.
